# Retrospective study of quadratus lumborum block for postoperative analgesia in patients undergoing percutaneous nephrolithotomy

**DOI:** 10.1186/s12871-020-01134-3

**Published:** 2020-08-31

**Authors:** Luning Chen, Jingjing Ji, Yali Tian, Qing Sun, Xuefeng Qiu, Xiaogong Li, Bingbing Li

**Affiliations:** 1 Department of Anesthesiology, Drum Tower Hospital Affiliated Nanjing University Medical School, Road 321#, Nanjing, Zhongshan, 210008 China; 2 Department of Surgery, Urology Surgery, Drum Tower Hospital Affiliated Nanjing University Medical School, Road 321#, Nanjing, Zhongshan, 210008 China

**Keywords:** Quadratus lumborum block, Percutaneous nephrolithotomy, Analgesia, Postoperative, Ultrasound-guided block

## Abstract

**Background:**

The postoperative analgesic effect of transmuscular quadratus lumborum block (QLB-TM) in patients following lower abdominal surgeries has been identified; however, the efficacy of QLB using the lateral approach (QLB-L) is still in debate. Therefore, this retrospective study was conducted to investigate the effect of a single-shot block with QLB-L on postoperative analgesia for patients undergoing percutaneous nephrolithotomy (PCNL).

**Methods:**

The medical information of the patients undergoing PCNL was retrieved from the electronic charter system (Medisystem, Suzhou, China) in our Nanjing Drum Tower Hospital during the period of Jan/2019 to Jun/2019. Among the total of 57 patients, there are 17, 18, and 22 patients subjected to QLB-L, QLB-TM, or routine treatment, respectively. The primary observational parameter was to assess postoperative pain with visual analog scales (VAS) at rest 30 min after extubation, 24 h, and 48 h after surgery, respectively. The secondary observatory endpoints, including the consumption of intraoperative opioids, the cumulative dose of non-steroid anti-inflammatory drugs (NSAIDs) and the incidence of adverse events related to postoperative analgesia, were evaluated as well.

**Results:**

The static VAS score at 24 h after surgery and the intraoperative consumption of sufentanil were significantly lower in patients receiving either intervention of QLB-L or QLB-TM as compared with those receiving routine treatment. However, one shot of QLB had no impact on VAS scores at 30 min post-extubation, 48 h after PCNL procedure compared with the patients receiving routine treatment. The percentage of non-ambulatory patients within 24 h post-PCNL was significantly higher in the QLB-TM group compared with the routine treatment group (*P* = 0.04). There were no significant differences in the incidence of postoperative nausea and vomit (PONV), itches, respiratory depression, the time for the first defecation, and the length of hospital stay (LOS) among the three groups.

**Conclusions:**

QLB-L procedure may exert as equivalent as QLB-TM in terms of abrogating postoperative pain within 24 h post-surgery and decreasing intraoperative sufentanil consumption in patients undergoing PCNL procedure as well. The caution should be taken to avoid lower extremities weakness in the patients after QLB-TM within the first 24 h post-PCNL procedure.

## Background

Percutaneous nephrolithotomy (PCNL) is the treatment of choice for patients with multiple or complex kidney or upper urinary tract stones, which necessitates the meticulous multi-modality analgesia due to mild to moderate pain originated from renal capsule dilation or nephrostomy-tube-related stress during the first 24 h after operation [1]. Hence, alternative options such as thoracic paravertebral block (TPVB) or quadratus lumborum block (QLB) technique are strongly recommended as an appropriate adjunctive to systemic intravenous analgesia for pain control [2–4]. In 2015, Børglum et al. defined transmuscular quadratus lumborum block (QLB-TM) using the Shamrock sign [5]. The injectate trajectory is aiming at the interfascial plane between the anterior border of quadratus lumborum (QL) muscle and psoas major muscle. Relative to the lateral approach of QLB (QLB-L) which was initially introduced by Blanco in 2007 and is only applied in limited clinical practices nowadays [6], the transmuscular approach has gained broader acceptance in a variety of abdominal surgeries comprising of cesarean section, renal, hernioplasty as well as laparoscopic procedures for its strong narcotic sparing effect [7–9]. The efficacy of QLB-L, as a variant of transverse abdominis plane (TAP) block, in postoperative pain control after PCNL was questioned for its incapability of providing the adequate analgesic plane from T9–12 [10]. The studies from Blanco and Kadam et al., however, suggested that QLB-L provided adequate postoperative analgesia in lower abdominal surgeries as well, given the local anesthetics were administered beneath the middle layer of the thoracolumbar fascia lateral to the QL muscle to acquire adequate cephalad-distribution with the blockade of T9–12 spinal nerves [11, 12].

In this study, we investigated the effect of ultrasound-guided QLB, targeting anterior-laterally or transmuscularly to the QL muscle, on pain relief after PCNL. Our hypothesis in this study is that the patients who receive QLB-L procedure record as low visual analog scales (VAS) value at rest as QLB-TM.

## Methods

### Ethical approval

This study was approved by the ethics committee of Drum Tower Hospital Affiliated with the Nanjing University Medical School (reference number: 2019–304-01).

### Methods

The medical information of the patients undergoing PCNL was retrieved from the electronic charter system (Medisystem, Suzhou, China) in our Nanjing Drum Tower Hospital from Jan/2019 to Jun/2019. Query used the following criteria: (a) anesthesia start time within the specified date/time parameters (Jan/2019-Jun/2019); (b) surgical procedure equal to PCNL;(C) patient has a completed pain assessment procedure form indicating the performance of QLB-L or QLB-TM. This is a retrospectively comparative study comparing the analgesic effect of ultrasound-guided QLB with two different approaches (QLB-L group, QLB-TM group) versus routine practice (control group) on postoperative pain following PCNL.

### Participants

Those eligible patients with age > 18 years old, American Society of Anesthesiologists (ASA) physical status classification I-III scheduled to undertake the selective PCNL procedure were included in this retrospective study. The patients with the history of severe hypertension (systolic blood pressure > 180 mmHg), diabetes mellitus with neuropathic pain, hepatic cirrhosis, renal function insufficiency, depressant condition, alcohol addiction, chronic intake of oral non-steroid anti-inflammatory drugs (NSAIDs) or analgesics, or with incomplete/loss of clinical data were excluded in this study.

### Anesthesia and perioperative management

The decision on which type of QLB approach performed before general anesthesia or intravenous medication alone without the requirement of truncal nerve block for pain control after PCNL was made by practitioners their own according to their daily routine of anesthetic management for those PCNL patients.

**Fig. 1:**
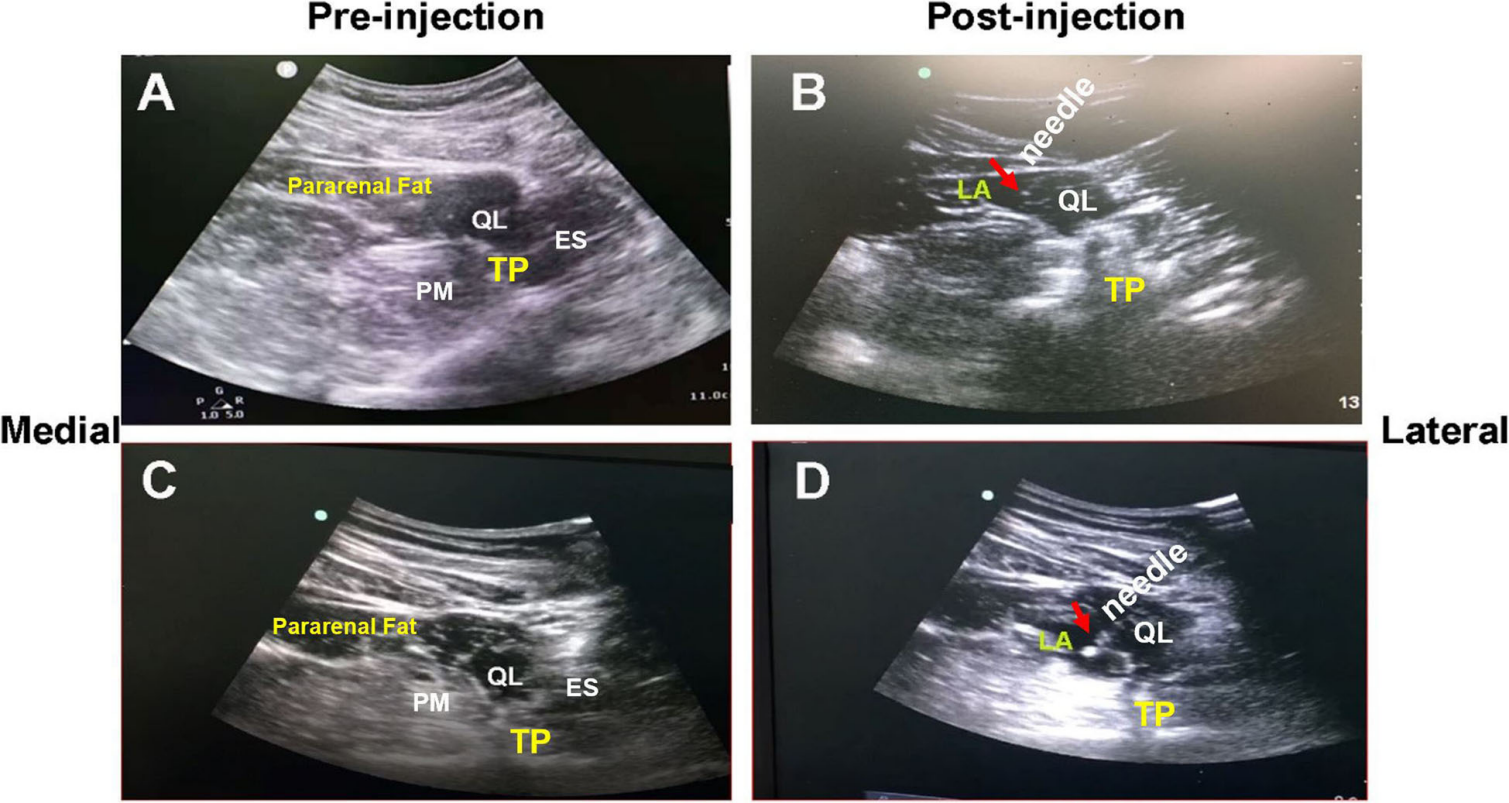
Schematic illustration of the procedure of QLB-L (**a**: Pre-injection; **b**: Post-injection of local anesthetics) and QLB-TM (**c**: Pre-injection; **d**: Post-injection of local anesthetics). QL: Quadratus lumborum; PM: Psoas major muscle; ES:Erector spinae muscle; TP: Transverse process; LA: local anesthetics; Red arrow: tip of the stimuplex needle®

QLB-L technique (schematic illustration in Fig 1a and b): The patient was placed securely in the lateral decubitus position. A low-frequency curvilinear probe (SonoSite Edge, transducer C60x/5 2 MHz, Fujifilm Sonosite Inc., US) was attached above the iliac crest. Under the guidance of ultrasound, the 18 gauge 10-cm needle (Stimuplex® D, B. Braun Medical Inc., Germany) was applied to puncture from dorsal to ventral direction aiming at the anterolateral margin of the junction of QL and transversalis fascia, and 5 ml saline solution was injected to confirm the correct position by hydro-dissection phenomenon [13]. The block was completed with the 0.375% ropivacaine at a volume of 0.5 ml/kg thereafter. The range of sensory block was tested 30 min after the procedure. Patients whose block plane of abdominal wall between T10-L1 level 30 min after the block were the eligible candidates receiving truncal nerve block technique for postoperative analgesia.

QLB-TM technique (Fig 1c and d): The patient was placed in the same position as the QLB-L technique. The ultrasound probe was vertically attached above the iliac crest. The transverse process of second lumbar vertebra (L2) and typical shamrock image were identified, and the needle was inserted from the edge of the probe and proceeded further into the fascia between the QLM and psoas major muscle. The local anesthetics were injected into the accurate interfascial plane between these two muscles.

All patients received standard general anesthesia monitoring, and intravenous infusion commenced with Lactate Ringer’s solution at the rate of 400 ml/h to replace the fluid loss owing to 8 h fasting and no drinking. Anesthesia was induced with intermittent bolus injection of (midazolam 0.01 mg/kg), propofol (2 mg/kg), sufentanil (0.2 microgram/kg), and cisatracurium (0.2 mg/kg) facilitated in tracheal intubation. The mechanical ventilation was commenced to maintain the end-tidal PCO_2_ at 40 mmHg. General anesthesia was obtained by total intravenous anesthesia with continuous infusion of propofol (4–6 mg/kg/min), cisatracurium (2 microgram/kg/min) to ensure appropriate sedation (BIS at the range of 40–60), substantial analgesia and muscle relaxation, respectively. The invasive arterial pressure monitor was established via left radial artery cannulation with a transducer connected to Philips (IntelliVue MP60, Bothell, Washington, United States) before surgery. Intermittent sufentanil (0.1 microgram/kg) was given if the heart rate (HR), blood pressure, or both increased more than 20% of the baseline. At 10 min before the beginning of the surgical procedure, 50 mg of flurbiprofen dissolved in 100 ml saline were intravenously administered. The hemodynamic parameters and oropharyngeal temperature were recorded automatically by Medisystem electronic charter database (Suzhou, China) in our department.

At the end of the operation, the patient was transferred to the post-anesthesia care unit (PACU), and the tracheal tube was removed after full emergence from anesthesia. The patient was discharged from the PACU to the ward once Steward Scale score (a, Awake degree: fully awake: 2 points; response to stimulation: 1 point; no response to stimulation: 0 point. b, Airway patency: can cough according to the doctor’s order: 2 points; can maintain airway patency without support: 1 point; respiratory tract need support: 0 point. c, Limb mobility: limbs can do conscious activities: 2 points; limbs can make unconscious activities: 1 point; no movement of limbs: 0 point.) was higher than five assessed by an experienced physician in PACU. The VAS method was used for postoperative pain intensity quantification with the scale ranges from 0 to10 (0/10: pain-free, 10/10: severe pain that participants could not tolerate). VAS scores were assessed by a well-trained doctor at 30 min immediately after extubation, every other 4 h thereafter till 48 h post-surgery in the ward. When the VAS score of patients was over 3, 50 mg flurbiprofen was titrated to free of pain. Given the VAS score over 5, dizocine (0.1 mg/kg) was administrated for pain control as a rescue dose. The maximal dose of flurbiprofen or dizocine is 200 mg, 0.2 mg/kg daily, respectively.

### Data collection

Preoperative demographic variables included the following: age (years); gender; BMI (body mass index: kg/m^2^); ASA classification; underlying diseases (Diabetes, Coronary artery disease, Hypertension). Intraoperative variables included the following: duration of operation (minutes); anesthesia time (defined as the time spent in the operating room in minutes); categories of drugs used for general anesthesia; intraoperative mean arterial pressure (MAP) and HR; dosage of intraoperatively administered narcotics. Postoperative variables included the following: additional analgesic requirement (NSAIDs, Dizocine); pain intensity assessed on VAS; the incidence of postoperative nausea and vomiting (PONV), itching, respiratory depression, the time for the first defecation, and the length of hospital stay (LOS).

### Outcome measurements

The primary outcome was VAS scores of patients at rest at 30 min post-extubation, 24 and 48 h after surgery, respectively. Secondary outcomes comprised of blood pressure and HR of patients recorded at the following time-points: arrival at the operating room (T0), beginning of surgery (T1), the end of surgery (T2), immediate post-extubation (T3), and leaving PACU (T4); the duration of operation, intraoperative opioid (sufentanil) consumption; LOS; postoperative additional analgesic requirement within 48 h after PCNL as well as the adverse effects (such as PONV, itching, respiratory depression, the time for the first defecation) were recorded for further analysis.

### Statistical analysis

Power analysis was based on results of the preliminary estimation of the difference in VAS at 24 h after PCNL procedure among various treatment groups from our electronic charter database; specifically, power was set at 0.8, significance criterion was set at 0.05, sigma was at 0.74. This yielded a sample size of 17 for each group. A Shapiro-Wilk test was used to evaluate whether or not the data are normally distributed; The distribution of each continuous variable was presented with s mean ± standard deviation or median with range (R)/ interquartile range (IQR). The distribution of each categorical variable was summarized in terms of its frequency and percentage. One-way analysis of variance using LSD correction for multiple comparisons of parametric variables and the Kruskal-Wallis test was used for the non-parametric variables followed by Dunn’s test for multiple comparisons. Repeated measurement analysis of variance was used to analyze repeated measurements. The test of chi-square or Fisher’s exact test was applied for the categorized data. A *P* value of < 0.05 was accepted as statistically significant. The analysis of the data was carried out using the IBM SPSS 21.0 statistical package software.

## Results

As is shown in Table 1, the demographic characteristics including gender, age, BMI, ASA physical status, comorbidities, and duration of surgical procedure of patients were recorded among three groups. There were no differences observed in preoperative or perioperative features among the three groups.

**Table 1:** Demographic characteristics, surgical data of the patients undergoing PCNL

Varibles	Con (***n*** = 22)	QLB-TM (***n*** = 18)	QLB-L (***n*** = 17)	F,Z or χ2	P
Age (years)	48.7 ± 10.0	55.1 ± 8.1	51.1 ± 11.2	2.16	0.12
Gender (M/F)	16/6	9/9	13/4	3.35	0.19
BMI (kg/m2)	26.9 ± 3.3	25.2 ± 3.3	25.6 ± 3.9	1.25	0.30
ASA (II/III)	15/7	12/6	11/6	0.05	0.97
Hypertension n(%)	5 (23%)	5 (28%)	5 (29%)	0.25	0.89
Diabetes n(%)	3 (14%)	3 (17%)	1 (6%)	1.00	0.60
Coronary disease n(%)	0 (0%)	0 (0%)	1 (6%)	2.40	0.30
Duration of Operation (min)	117.6 ± 40.2	114.4 ± 40.4	116.6 ± 44.8	0.03	0.96

Data are presented as mean ± standard deviation, case (%)

*BMI* Body mass index, *M* Male, *F* Female, *ASA* American Society of Anesthesiologists

*Con* Control, *QLB-L* QLB-Lateral, *QLB-TM* QLB-transmuscular

**Fig. 2:**
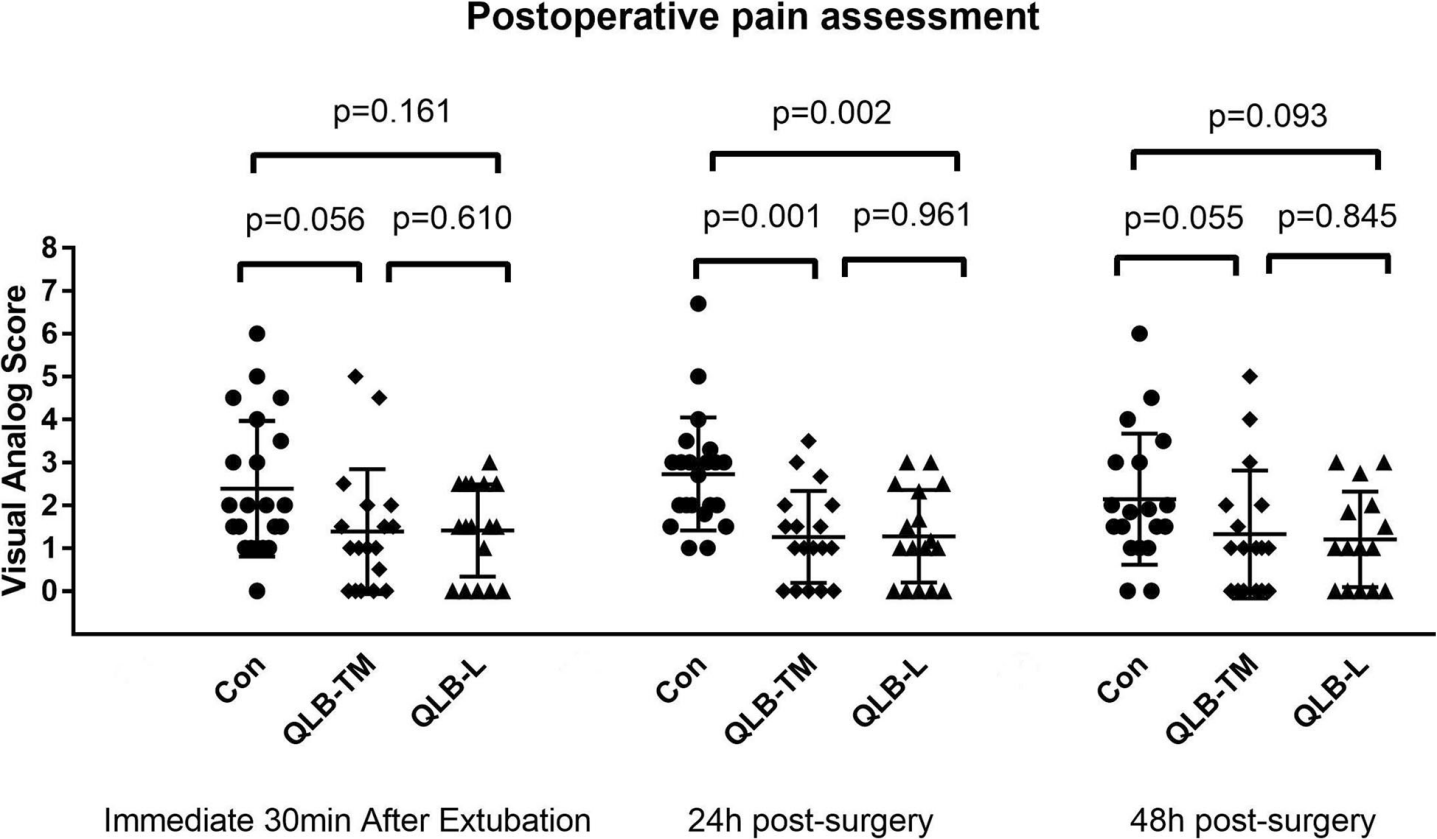
Effect of either type of QLB on the VAS scores in patients undergoing PCNL. The VAS scores were assessed at 30 min immediately after extubation (**a**), 24 h (**b**), and 48 h after surgery (**c**). The data was presented with mean ± standard deviation if it conformed to normal distribution or median with the range/ interquartile of range if not normally distributed. Con: control; QLB-L: quadratus lumborum –lateral; QLB-TM: quadratus lumborum- transmuscular

The postoperative pain scores assessed using VAS were collected at 30 min after removal of the endotracheal tube in a PACU, 24, 48 h after surgery in the ward, respectively. The patients receiving either type of QLB had lower VAS values at rest 24 h after surgery compared with the patients under routine analgesic treatment (QLB-TM vs Con: 1(0–2) vs 2(1.9–3.1), *P* < 0.01; QLB-L vs Con: 0(0–1) vs 2(1.9–3.1), *P* < 0.01). As shown in Fig. 2, however, at the time-point of 30 min post-extubation or 48 h after surgery, there is no significant difference in VAS values among three groups. The pain intensity for each group at different timepoints were shown in the supplementary material (Supplementary Table 1) .

**Fig. 3:**
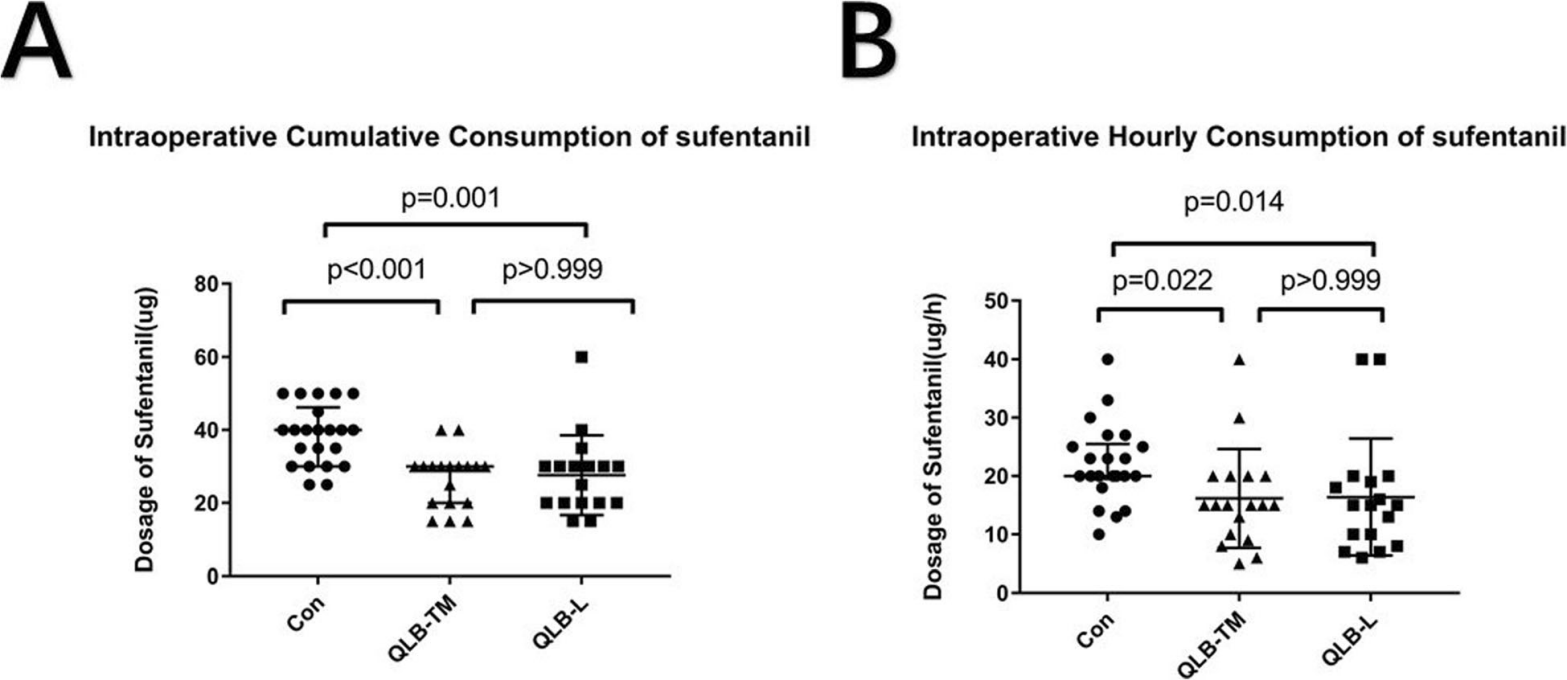
Effect of either type of QLB on the intraoperative sufentanil consumption (**a**: cumulative dosage; **b**: the dosage of sufentanil consumed per hour in patients undergoing PCNL). The data was presented with the median with an interquartile of range. Con: control; QLB-L: quadratus lumborum –lateral trajectory; QLB-TM: quadratus lumborum- transmuscular trajectory

**Fig. 4:**
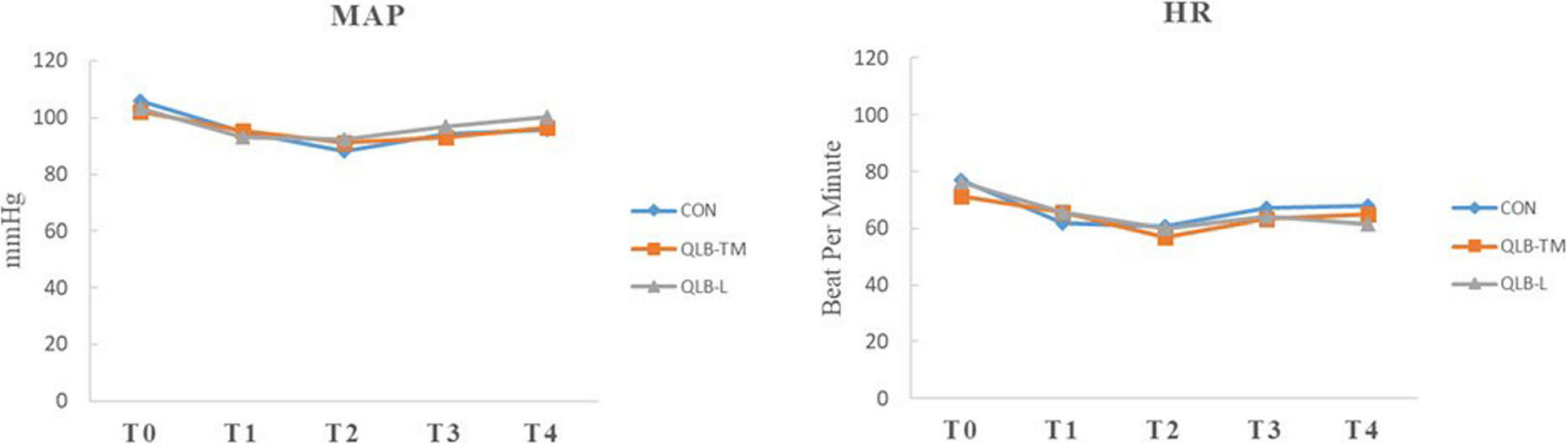
Effect of either type of QLB on the perioperative mean arterial pressure (MAP) and heart rate (HR). The MAP and HR were recorded at arrival at the operating room(T0), the beginning of operation(T1), the end of the operation(T2), extubation(T3), and transfer from PACU(T4). The data are presented as mean ± standard deviation. Con: control; QLB-L: quadratus lumborum –lateral; QLB-TM: quadratus lumborum- transmuscular

As shown in Fig. 3, the intraoperative cumulative consumption (Fig 3a) or the consumption dosage/per hour of sufentanil (Fig 3b) was 40.0 (30.0–48.8) microgram, 20.0 (19.0–26.0) microgram /h in control group respectively, which was higher compared with either of QLB group respectively (*P* < 0.05). The results of each group were shown in the supplementary material (Supplementary Table 2). As shown in Table 2, the median value of the cumulative dose of NSAIDs during the first 24 h after surgery was (75 (0–150) mg) in QLB-TM group and (50 (0–100) mg) in QLB-L respectively, which was equivalent to that consumed in the control group. Furthermore, there is also no significant difference in the rescue dose of dizocine among three groups within 48 h after PCNL. As shown in Table 3, either the first time of flatus or LOS was not shortened in patients receiving QLB treatment. As shown in Fig. 4, the application of either type of QLB had a negligible impact on the perioperative MAP and HR. The results of each group were shown in the supplementary material (Supplementary Table 3).

**Table 2:** Postoperative Consumption of flurbiprofen (mg)

Varibles	Con (***n*** = 22)	QLB-TM (***n*** = 18)	QLB-L (***n*** = 17)	F,Z or χ2	P
24 h NSAIDS	50 (0–150)	75 (0–150)	50 (0–100)	0.75	0.69
48 h NSAIDS	0 (0–100)	0 (0–100)	0 (0–100)	0.04	0.98
Dizocine rescue n(%)	1 (5%)	1 (6%)	1 (6%)	0.04	0.98

Data are presented as median (interquartile range)

**Table 3:** Recovery of patients following PCNL and adverse events

Varibles	Con (***n*** = 22)	QLB-TM (***n*** = 18)	QLB-L (***n*** = 17)	F,Z or χ2	p
LOS (days)	7 (6–10)	8 (7–9)	7 (5–8)	4.0	0.14
First time to Defecation (hours)	48 (48–60)	48 (36–60)	48 (36–48)	3.9	0.14
PONV n(%)	1 (5%)	1 (6%)	1 (6%)	0.4	0.98
Itches n(%)	0 (0%)	0 (0%)	0 (0%)		1
Respiratory depression n(%)	0 (0%)	0 (0%)	0 (0%)		1

Data are presented as median (interquartile range), case (%)

*LOS* Length of hospital stay (days) *POD* Postoperative day

*PONV* Postoperative nausea and vomiting

**Fig. 5:**
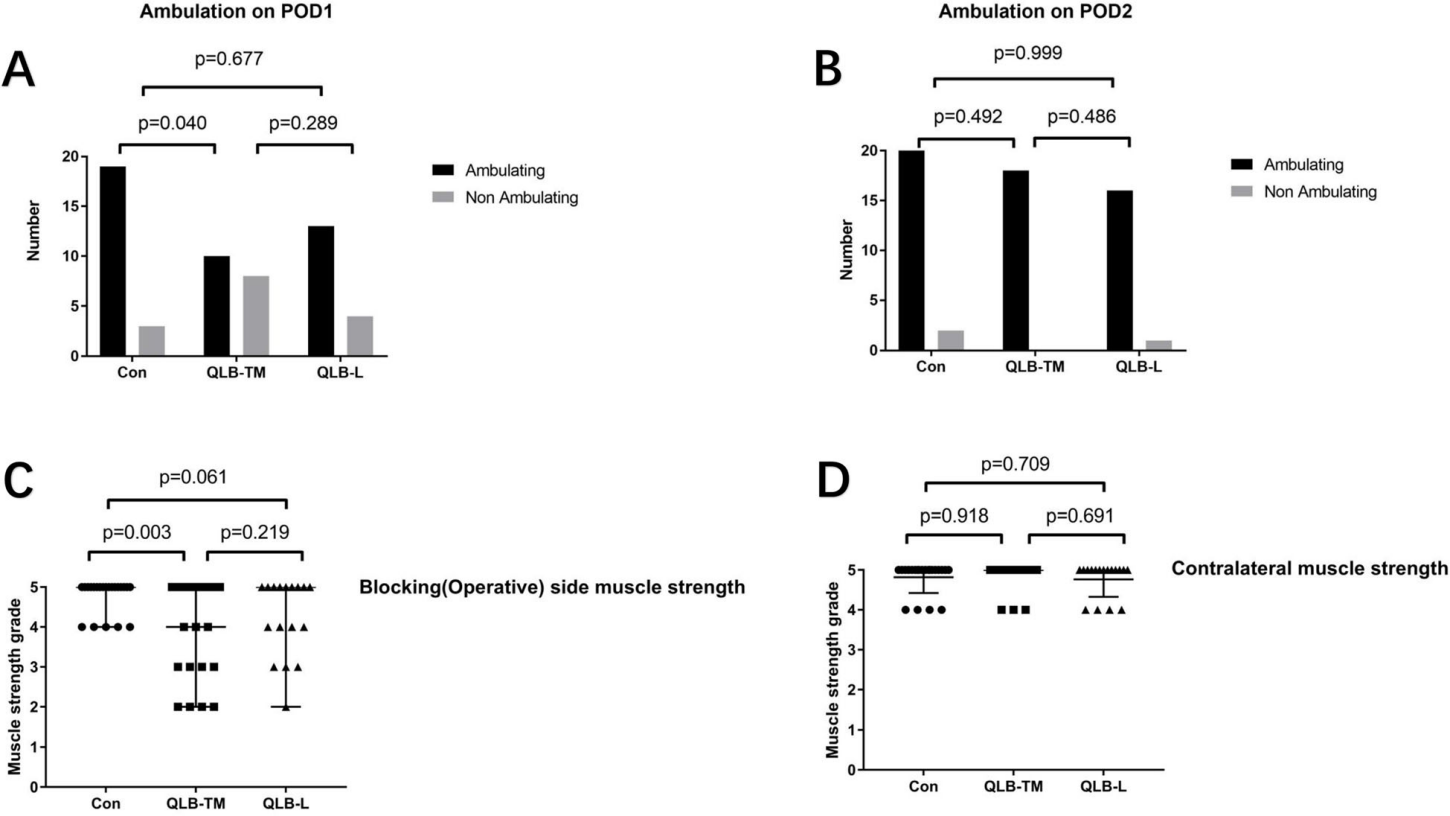
Effect of either type of QLB on the postoperative motor function of lower extremities and ambulation. The number of ambulatory and non- ambulatory patients on POD 1 (**a**), POD 2(**b**). Muscle strength score in blocking (Operative) side (**c**) or the contralateral side of the block (**d**). The ordinal data was presented median with the range/ interquartile of range. The test of chi-square or Fisher’s test was applied for the categorized data. Con: control; QLB-L: quadratus lumborum –lateral; QLB-TM: quadratus lumborum- transmuscular; POD: postoperative day

Consistent with the previous results, the incidence of PONV, itching, respiratory depression were not significantly different among the three groups (Table 3). In this study, we found that there were 8 out of 18 patients in the QLB-TM group who could not get out of bed and ambulated voluntarily without the assistance of an instrument during the first 24 h after surgery, the percentage of which was statistically higher compared with the Con group (*P* = 0.04, Fig. 5a). The lower extremity muscle strength score (Muscle strength grade: grade 0: in a state of complete paralysis, while the muscles cannot contract; grade 1: the muscles can contract, but cannot complete the action; grade 2: the limbs can move in parallel on the bed, but cannot lift off the bed surface; grade 3: the limbs can be raised to leave the bed surface, but cannot resist resistance; level 4: the limbs can resist part of the resistance; grade 5: the muscle strength is normal.) in the ipsilateral side of the block was lower in the QLB-TM group compared with the Con group on postoperative day 1 (POD 1) (*P* = 0.003, Fig. 5c). However, the ratio of the ambulatory patients to all subjects and muscle strength scores in the ipsilateral side of the block in QLB-L group 24 h post-PCNL was not significantly different from Con group (*P* = 0.677, Fig. 5a and c). The lower extremity score in the contralateral side of the block was not significantly different among the three groups on POD 1 (Fig. 5d). Furthermore, the percentage of ambulatory patients on POD 2 was not significantly different among the three groups (Fig. 5b).

## Discussion

Our results in the present retrospective study suggest that QLB-L is as effective as QLB-TM in attenuating postoperative pain within 24 h after PCNL. Additionally, either type of QLB procedure similarly decreases the intraoperative consumption of sufentanil without causing hemodynamic instability, albeit it could not lead to further decrease in the incidence of adverse events associated with narcotics use and shortening of LOS.

There are at least four types of QLB approaches performed in the clinic at present. Except for the intramuscular technique reported by Murouchi [14], the other 3 QLB techniques are used by the majority of physicians for pain control following diverse types of surgeries [15]. Actually, QLB-TM has been accepted as one of the most popular approaches in abdominal surgeries as an adjunctive to systemic analgesia, bearing better and reliable pain control advantage [16]. Albeit these distinct approaches of QLB (posterior, lateral, and transmuscular) may have different analgesic efficacy, the quantitative analysis of the analgesic potency of these approaches yet lacks due to the heterogeneity in surgical types [17]. The first prospective study was conducted by Ahmed et al. in the year of 2019 to compare the analgesic efficacy in patients receiving either posterior or transmuscular approach of QLB in unilateral inguinal hernia repair [18]. Their results implicated that QLB-TM is superior to the posterior approach in decreasing the postoperative pain intensity. In the recent two prospective clinical studies conducted by Dam and KılıçE et al. respectively [10, 19], the potency of transmuscular approach of QLB in postoperative pain relief after PCNL procedure was assessed. As a result, QLB-TM rendered the beneficial effect in alleviating the pain of patients during activity after surgery, shortened the time to ambulation, LOS, and promoting the recovery of patients. In line with the previous studies, QLB-TM elicited significant pain relief at rest 24 h-post PCNL in the present study. Furthermore, all patients had an analgesic plane from T9 - L1 30 min after QLB-TM prior to general anesthesia induction and played a role in decreasing the intraoperative sufentanil consumption owing to the blockade of noxious afferent signal from the surgical site.

Nevertheless, there is still no comparison between the transmuscular and lateral approach of QLB after PCNL surgery in a randomized prospective study until now. Our results in this retrospective study showed that the loss of cold sensation or pinprick dermatome plane 30 min after QLB-L was not distinct from the QLB-TM group at the extent from T9 to L1, which provided satisfactory intraoperative analgesia and was facilitated to decreasing the sufentanil consumption. Besides, the analgesic effect of QLB-L was equivalent to that of QLB-TM within 24 h post- PCNL surgery in the present study. In the study by Dam et al., they reiterated the importance of the accurate position of needle at the plane between the anterior border of quadratus lumborum muscle and psoas muscle to ensure the cranial spread of local anesthetics beyond arcuate ligament into the thoracic paravertebral region [11]. The trajectory in the lateral approach of QLB, they thought, was directed the fascial plane between the pararenal fat layer and middle thoracolumbar fascia, which prevented local anesthetics entering thoracic paravertebral spaces and barely had the value of clinical significance in pain control for PCNL patients. However, consistent with several other studies [20], our results demonstrate that the lateral type of QLB exerts a favorable effect in controlling postoperative pain after PCNL with the duration of analgesia lasting as long as 24 h. The mechanism underlying the analgesic effect of QLB-L for PCNL patients in the present study is still elusive. The plausive explanations can be attributed as follows: first, the lateral QLB is inclined to spread cephalad beneath the middle layer of thoracolumbar fascia and transversalis fascia in anatomy to interfere the afferent pain signal of T9–12 spinal nerves, which had been identified by several clinical or cadaver studies [15]. Additionally, the injection of 0.5 ml/kg (0.375% ropivacaine) was a relatively larger volume than that used in the previous studies and might be facilitated in the cranial spread of the drug.

We observed that the number of non-ambulatory patients in the QLB-TM group on POD 1 approximated 2-fold of that in the conventional treatment group. The weakness of lower extremities was identified in patients receiving QLB-TM. Previous studies indicated that the QLB-TM reduced postoperative 24 h pain scores and opiates consumption in patients after total hip arthroplasty [21]. The lower extremities weakness was accidentally reported in patients receiving QLB-TM [22]. Recent cadaver study conducted by Carline to assess the dimension of stained nerves using the following three different QLB groups [15]. They observed that a higher rate of lumbar plexus stain (L1–3) occurred in the QLB-TM group. The injection site in the vicinity of lumbar plexus and local anesthetics infiltration accounted for the faintness in lower extremities and better pain control after hip arthroplasty. The LOS was not significantly shortened in QLB groups compared with the routine control group in the present study. One shot of injection has only 24 h duration of pain relief after PCNL; therefore, the advantage of QLB on controlling pain is not evident contrary to the continuous QLB strategy in pain control after liver surgery [23].

There are still several limitations in this study: Given this small size preliminary retrospective cohort study, hence a prospective randomized study is warranted to assess the beneficial pain control effect of QLB-L with ropivacaine compared with placebo in PCNL patients. The larger sample size is needed to detect the difference in lower extremity weakness 24 h after PCNL among QLB groups and the difference in LOS between QLB and routine intervention. Lastly, the optimal concentration and volume for ropivacaine for unilateral QLB are still unknown, and our study cannot give information regarding the adequate dose or volume of QLB in postoperative analgesia.

## Conclusions

Collectively, ultrasound-guided QLB-L technique may provide as similar satisfactory pain-control as QLB-TM after PCNL. Cautions should be taken in patients for the latent risk of the weakness of low extremities during the first 24 h after QLB with the transmuscular approach, which can be avoided by being substituted with the lateral approach. Nevertheless, in view of this small-sample retrospective study, high-grade evidence is needed to confirm with confidence the analgesic effect of QLB-L for PCNL patients.

## Supplementary information

**Additional file 1:** **Table S1.** Comparison of postoperative pain (VAS). **Table S2.** Intraoperative Consumption of Sufentanil (microgram). **Table S3.** Comparison of mean arterial pressure (MAP) and heart rate (HR)

## Data Availability

The datasets used and/or analyzed during the current study are available from the corresponding author on reasonable request.

## Abbreviations

ASA: American Society of Anesthesiologists
BMI: Body-mass index
HR: Heart rate
LOS: Length of hospital stay
MAP: Mean arterial pressure
NSAIDs: Non-steroid anti-inflammatory drugs
PACU: Post-anesthesia care unit
PCNL: Percutaneous nephrolithotomy
POD: Postoperative day
PONV: Postoperative nausea and vomit
QL: Transmuscular quadratus lumborum block
QLB: Quadratus lumborum block
QLB-L: Lateral approach quadratus lumborum block
QLB-TM: Transmuscular quadratus lumborum block
TAP: Transverse abdominis plane block
VAS: Visual analog scale
