# Supplementary material for: Retrospective study of quadratus lumborum block for postoperative analgesia in patients undergoing percutaneous nephrolithotomy
Source: BMC Anesthesiol. 2020 Aug 31;20:217. doi: 10.1186/s12871-020-01134-3 (PMC7457541; doi:10.1186/s12871-020-01134-3)
Supplement: Supplementary file 1 — Additional file 1: Table S1. Comparison of postoperative pain (VAS). Table S2. Intraoperative Consumption of Sufentanil (microgram). Table S3. Comparison of mean arterial pressure (MAP) and heart rate (HR) [file 12871_2020_1134_MOESM1_ESM.pdf]

**Table 1.**	Comparison of postoperative pain(VAS)			
**Time**	**Con (n=22)**	**QLB-TM (n=18)**	**QLB-L (n=17)**	**P**
T0	2(1-3.6)	1(0-2)	1.5(0-2.5)	0.122
	(1.6,3.0)	(0.6,2.2)	(0.9,2.0)	
T1	2(1.5-3.3)	1(0.8-2.3)	1.6±1.1	0.148
	(1.8,3.0)	(0.8,2.2)	(0.2,2.1)	
T2	2(1.5-3.6)	1(0.8-2)*	1(0-1.5)*	<0.01
	(2.1,3.3)	(0.7,1.8)	(1.0,1.9)	
T3	3(2-3)	1.4±1.0*	0.5(0-1)*	<0.01
	(2.3,3.5)	(0.9,1.9)	(0.8,1.9)	
T4	2(1.9-3.1)	1(0-2)*	0(0-1)*	<0.01
	(2.1,3.2)	(0.6,1.7)	(0.7,1.8)	
T5	3(2-3)	1.2±0.8*	0.5(0-1)*	<0.01
	(2.2,3.1)	(0.8,1.6)	(0.8,1.7)	
T6	2.8±0.9	1(1-1.6)*	1.3±0.8*	<0.01
	(2.3,3.2)	(0.9,1.5)	(0.9,1.7)	
T7	2.2±1.3	1.1±0.8	1.4±0.8	0.103
	(1.6,2.8)	(0.7,1.5)	(0.9,1.8)	
T8	2.2±1.5	1(0-2.5)	0(0-1)	0.094
	(1.4,2.9)	(0.6,2.3)	(0.6,1.8)	

The data was presented mean ±standard deviation if it conformed to normal distribution or median (interquartile range) if not normally distributed, 95% CI in parentheses below. T0: 30 min immediately after extubation, T1: 4 p.m on the Day of Surgery, T2: 8 p.m. on the Day of Surgery, T3: 8 a.m on the POD 1, T4: 12 a.m. on the POD 1, T5: 4 p.m. on the POD 1, T6: 8 p.m. on the POD 1, T7: 8 a.m. on the POD 2, T8: 12 a.m. on the POD 2. *: Comparison of VAS at the same time point with the control group,* $p< 0.05$. VAS: visual analog scale, POD：postoperative day

Table 2. Intraoperative Consumption of Sufentanil (microgram)

Varibles	Con (n=22)	QLB-TM (n=18)	QLB-L (n=17)	P
Cumulative	40.0(30.0-48.8)<br>(35.0,42.3)	30.0(20.0-30.0) ***<br>(22.9,30.5)	30.0(20.0-30.0)**<br>(22.0,33.3)	<0.0001
Hourly	20.0(19.0-26.0)<br>(19.0,25.1)	15.0(9.8-20) *<br>(12.0,20.4)	15.0(9.0-19.5) *<br>(11.3,21.6)	0.005

The data was presented mean ±standard deviation if it conformed to normal distribution or median (interquartile range) if not normally distributed, 95% CI in parentheses below.

:Comparison with the control group, p< 0.05; **p<0.01;***p<0.001

Table 3. Comparison of mean arterial pressure(MAP) and heart rate(HR)

Time	Varibles	Con (n=22)	QLB-TM (n=18)	QLB-L (n=17)	F,Z or $\chi2$	P
T0	MAP	106.1±10.7	102.0±11.9	103.3±11.6	MAP:1.697	0.113
	HR	77.0±11.8	71.4±10.4	76.2±12.7		
T1	MAP	94.9±10.9	95.2±16.7	93.0±13.5		
	HR	61.8±9.1	65.7±6.2	65.8±12.8		
T2	MAP	88.1±9.5	91.3±8.9	92.5±8.0		
	HR	60.8±10.0	56.9±9.1	59.9±8.7		
T3	MAP	94.4±11.7	93.1±11.7	96.8±13.9	HR:1.611	0.131
	HR	67.2±10.6	63.4±13.1	64.2±12.7		
T4	MAP	95.7±10.7	96.6±9.2	100.2±11.8		
	HR	68.0±10.5	64.8±10.7	61.6±17.0		

Data are presented as mean±standard deviation

PACU: Postanesthesia care unit

T0: Arrival at the operating room;T1: The begining of operation;T2: The end of operation

T3: Extubation;T4: Transfer from PACU
